# Beyond counting: how single-cell long-read sequencing turns transcriptome complexity into precision targets

**DOI:** 10.3389/fonc.2026.1800370

**Published:** 2026-05-28

**Authors:** Ashley Byrne, Colette Felton, William Stephenson

**Affiliations:** 1 Department of Proteomic and Genomic Technologies, Genentech, South San Francisco, CA, United States; 2 Department of Biomolecular Engineering, University of California, Santa Cruz, Santa Cruz, CA, United States

**Keywords:** alternative splicing, driver variants, long-read sequencing, precision oncology, single cell, spatial sequencing, transcriptomics

## Abstract

Single-cell RNA sequencing (scRNA-seq) has emerged as a critical tool in oncology research, revealing key aspects of immune infiltration, tumor heterogeneity, and the tumor microenvironment. However, most scRNA-seq experiments capture only a portion of the 5’- or 3’-end of the gene due to limitations in sequencing read length. This limits short-read scRNA-seq to a method that quantifies gene expression but falls short of understanding the full complexity of the transcriptome. Since single nucleotide variants (SNVs), structural variants (SVs), and aberrant splicing are known drivers of tumor development, it is critical to be able to understand their heterogeneity at the single cell level. Long-read RNA-seq is capable of sequencing full-length molecules, simplifying the process of identifying these types of alterations. This review examines how single-cell long-read sequencing (scLRS) technologies are overcoming the limitations of short-read platforms to resolve the complexity of the cancer transcriptome. We highlight key applications that leverage full-length information, including the identification of novel tumor-specific neo-antigens and fusion genes. By linking genotype information with transcript expression, this technology holds the potential for developing highly specific, isoform-selective therapies that minimize off-target effects. We also describe the application of scLRS to sensitively trace tumor clone subtypes using isoform profiles and to identify clonal evolution through tracing SNV variation within single cells. Furthermore, we discuss the current state of the scLRS field and how it can be applied for multimodal analysis, which integrates full-length transcriptomics with genomic, spatial and proteomic data to create a more comprehensive profile of the tumor micro-environment. Finally, we outline the current technological and computational challenges of scLRS, including cost, throughput, and the need for standardized bioinformatic tools, providing a roadmap for future advancements. As these limitations are overcome, we foresee scLRS as an indispensable tool for uncovering the transcriptomic complexity within the tumor microenvironment, accelerating the development of precision oncology therapies.

## 1 Introduction

Cancer is a disease of adaptation characterized by a relentless ability to evolve and evade therapeutic pressure. Intratumoral heterogeneity and genetic diversity within cancer cells make it difficult to reliably eliminate the entire cancer cell population. The emergence of single-cell RNA sequencing (scRNA−seq) technology has driven a paradigm shift across the cell biology field, providing a higher resolution lens to uncover dynamic biological processes across a spectrum of diseases, including cancer (1–3). Although bulk RNA-seq can illuminate the global RNA landscape and gene regulation, it inherently masks cell type-specific contributions which are obscured by the statistical average of millions of cells. As a result, scRNA-seq has become the tool of choice in unraveling complexities within tissues due to its capacity to map cell types, trace complex developmental and differentiation trajectories, and identify rare, functionally-distinct cellular subpopulations.

**Figure 1:**
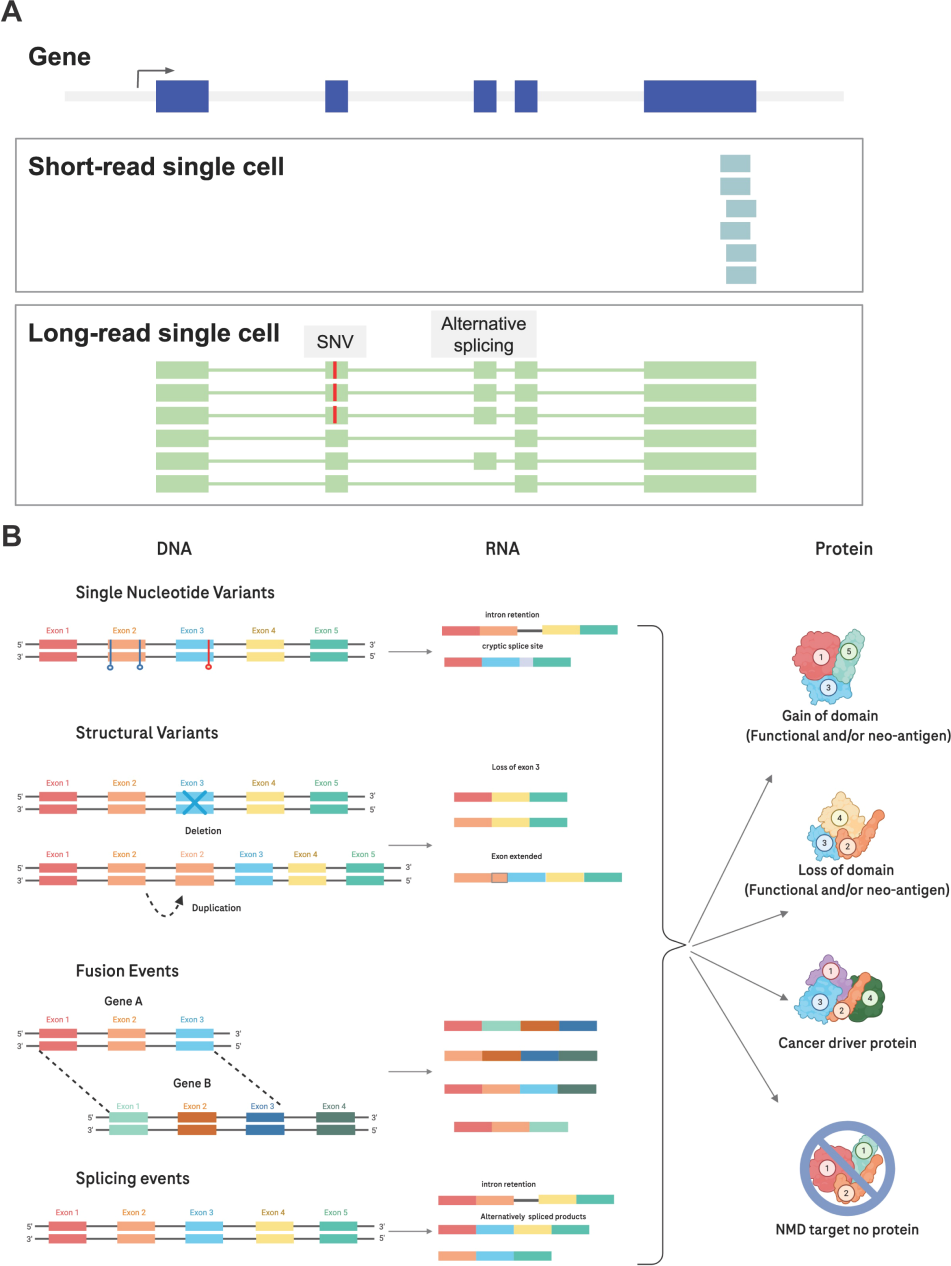
scLRS provides increased functional information compared to short-read single-cell RNA-seq. **(A)** Schematic showing droplet based single cell approaches sequenced on short-read platforms can only capture the 3’ end of transcripts. scLRS combined with droplet based approaches can produce reads that represent the entire transcript, including SNVs and alternative splicing. **(B)** Genetic variants and resulting splicing alterations in cancer can modify protein products, including altering protein domains and the generation of neo-antigens, making them valuable targets for cancer therapeutics. Schematic showing the different events that can be detected with scLRS and their functional outcomes. Created in BioRender. Byrne, A (2026). https://BioRender.com/f7q7uc0.

While scRNA-seq has successfully advanced our comprehension of cancer biology by clarifying the tumor microenvironment, its reliance on short-read sequencing restricts the analysis to gene-level expression. This limitation obscures critical layers of the transcriptional complexity, primarily due to most conventional droplet-based scRNA-seq platforms only capturing the 3’ or 5’ ends of a gene (**Figure 1A**) (4). The transcriptome consists of RNA isoforms that are generated through alternative splicing, alternative transcription start site usage, and alternative polyadenylation. These isoforms encode functional diversity, often dictating protein structure, localization, and activity—features frequently leveraged by cancer cells to drive progression and resistance (5, 6). Given that the vast majority of protein-coding genes express multiple proteoforms and the transcriptome is frequently dysregulated in cancer, we are compelled to ask how we can leverage this diversity for more precise targeting and development of cancer therapeutics.

Adopting third-generation long-read sequencing platforms, such as those developed by Pacific Biosciences (PacBio) and Oxford Nanopore Technologies (ONT), offers a solution to overcome this limitation and deepen our understanding of the transcriptome. These sequencing technologies generate reads that can span entire molecules, ranging from thousands to over a million base pairs. Long-read sequencing platforms have been adapted for single-cell capture approaches, designated as single-cell long-read sequencing (scLRS). Initial single-cell long-read sequencing efforts, using ONT and PacBio only evaluated a few cells (7, 8). However, this rapidly evolved to looking at thousands of single cells using the droplet based approach (9, 10). These early foundational studies demonstrated that scLRS can accurately recapitulate gene expression, comparable to established scRNA-seq methods, while also resolving complex splicing events at the single cell level (7). This technology not only illuminates isoform diversity within cell types, but can significantly improve the resolution of cancer cell genetic diversity.

**Figure 2:**
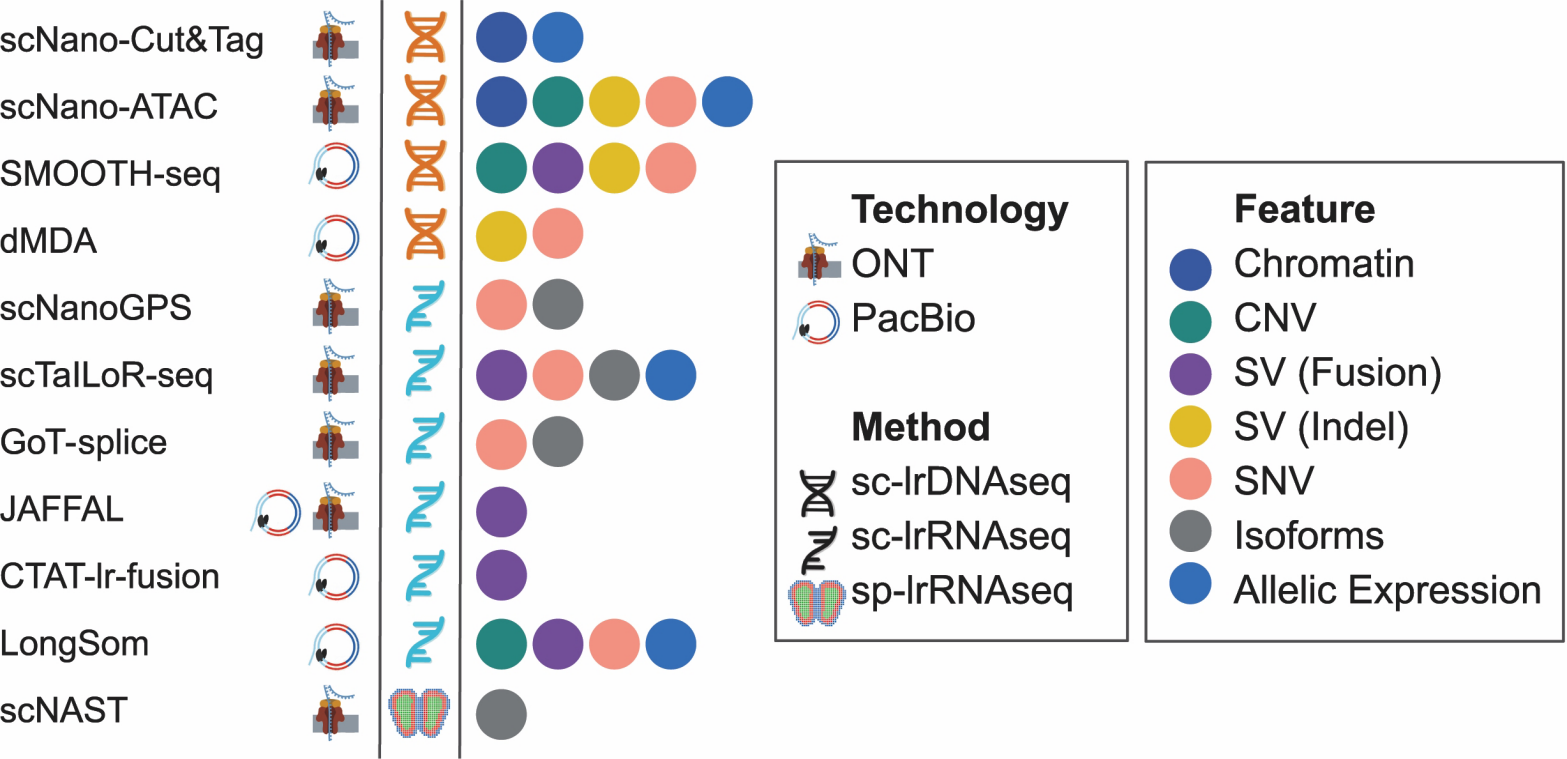
scLRS cancer studies showing demonstrated features detected.

Genomic alterations and splicing events derived from cancer cells can have a profound, direct consequence on protein products. This may result in aberrant proteins that function as effective targets (**Figure 1B**), which can subsequently be exploited for precision-based therapeutics. The capacity to detect genomic alterations such as single nucleotide variants (SNVs), structural variants (SVs), gene fusions, and allelic expression information remains constrained in current short-read scRNA-seq investigations (11). Currently, scLRS tools are being developed to utilize SNVs as “natural barcodes,” thereby allowing researchers to delineate cell lineage, track the evolution of distinct cancer subclones (such as in Acute Myeloid Leukemia or Chronic Lymphocytic Leukemia), and reveal cell-type-specific mutation patterns within the tumor microenvironment (12–14). Structural variants, including gene fusions, within-gene domain deletions, duplications, and inversions, can now be detected at the single-cell level, facilitating a more sensitive analysis of tumor evolution (15). By integrating precise genotypic and transcriptomic features within the same cell, we can significantly increase the resolution of overall tumor cellular heterogeneity. This enhanced resolution is crucial for tracking tumor cell co-evolution, offering a clear translational benefit for both diagnosis and therapeutic decision-making. For a comprehensive summary of the current scLRS methodologies and tools that detect these specific transcriptomic or genomic features within cancer samples see **Figure 2**.

RNA isoform biology is intricately tied to several cellular processes. Splicing occurs co-transcriptionally, meaning that chromatin conformation, histone modifications, DNA methylation, and promoter selection can shape splice-site choice, alternative transcription start site (TSS) usage, and untranslated region (UTR) selection before transcripts have even disengaged from the transcriptional machinery (16, 17). Splicing and alternative transcription can lead to proteoforms with distinct functions or protein/protein interactions within cells. Analyzing RNA isoforms and their complete transcript structure—including UTR regions, alternative start/end site usage (TSS/TES), splicing, and allele-specific expression—offers critical insights into the proteome, the transcriptome, and the overall health of the genome. Measuring full-length isoforms using scLRS provides a uniquely informative readout, especially when combined with orthogonal modalities that measure chromatin accessibility, protein expression, or are analyzed in a spatial context. Here, we discuss how integrating these emerging technologies with scLRS creates a powerful framework that has the potential to be a vital resource for discovering tumor-specific splicing targets of clinical significance (18).

## 2 Applications in precision oncology

### 2.1 Resolving the cancer transcriptomic landscape: isoforms, proteome expansion, and neo-antigens

Transcriptome diversity drives proteome expansion, primarily influenced by alternative splicing, transcription initiation, and alternative polyadenylation cleavage events. Global splicing analysis across different cancers has shown that dysregulated splicing facilitates the production of noncanonical and cancer-specific mRNA transcripts that can either inactivate tumor suppressors or activate oncogenes, thereby initiating cancer signaling pathways (19). Consequently, this dysregulation is a major contributor to nearly all hallmarks of cancer, including sustained proliferation, cell invasion, metastasis, and stemness (20–22).

One way cancer cell transcriptomes can be dysregulated is through isoform imbalance. This imbalance is primarily driven by alternative splicing or promoter selection changing the ratio of isoforms expressed in cancer cells promoting tumor growth, metastasis, and drug resistance. Aforementioned distinct protein isoforms can lead to different functions or even possess antagonistic biological functions that when left unchecked can contribute to cancer progression. One classic example is the splicing of the BCL2L1 gene, a known apoptosis regulator. BCL2L1 mainly forms two antagonistic isoforms: BCL-xS (pro-apoptotic) and the BCL-xL (anti-apoptotic);when the balance between these isoforms is skewed it drives tumorigenesis (23). Similarly, alternative splicing of the gene CD44, (which encodes a transmembrane glycoprotein known to be involved in cell division) can produce the pro-oncogenic CD44s isoform which drives the epithelial-to-mesenchymal transition in breast and ovarian cancers when dominantly expressed (24, 25).

A significant benefit of long-read sequencing is its remarkable capacity to uncover previously unannotated novel transcripts, in addition to pinpointing isoform switching events. For instance, long-read sequencing has led to the discovery of thousands of previously unannotated isoforms in various cancers, including studies identifying novel transcripts in breast cancer and hepatocellular carcinoma (26, 27). Importantly, many of these novel transcripts affect protein-coding sequences, leading to altered protein localization and function.

**Figure 3:**
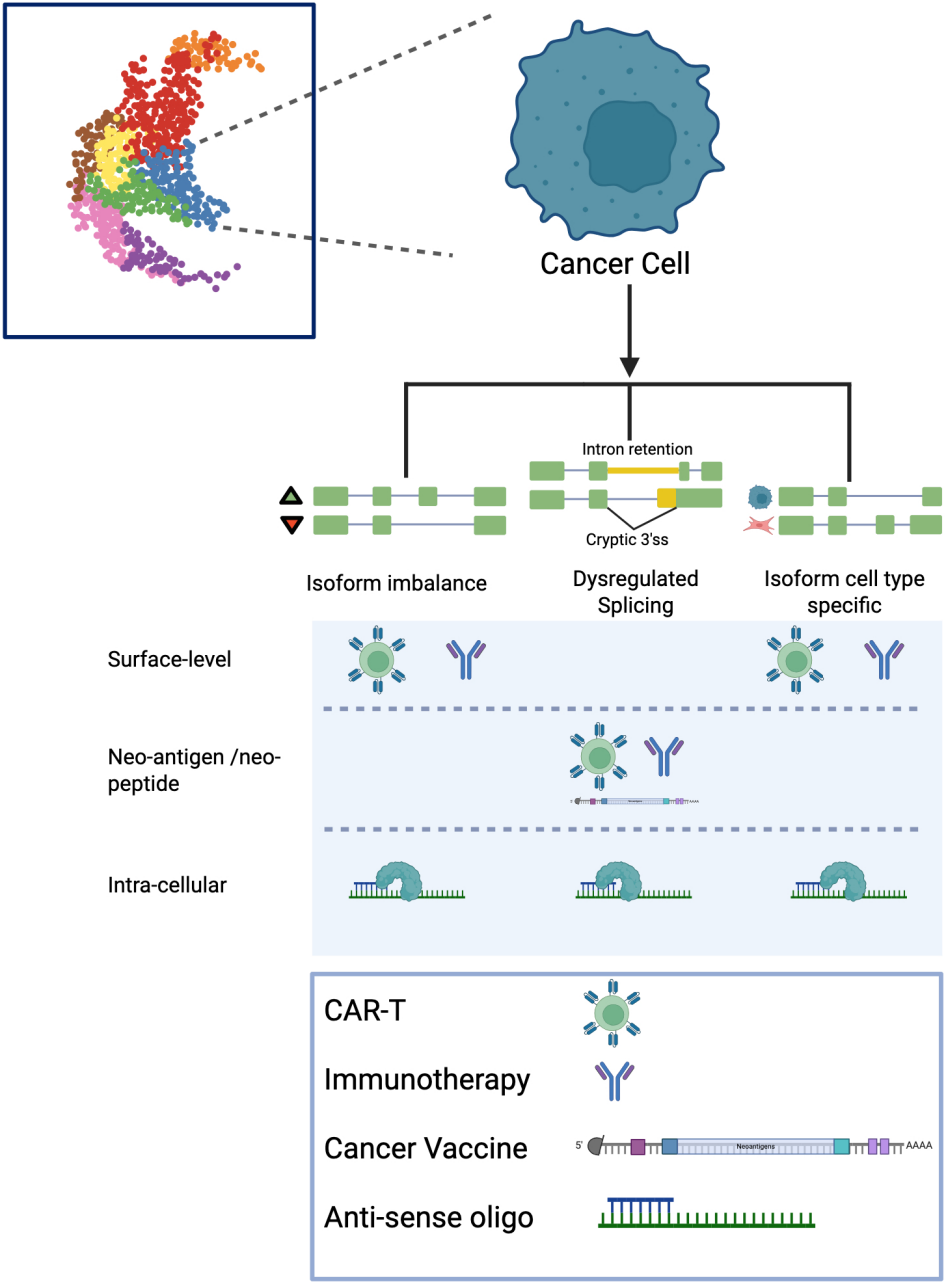
Therapeutic strategies for targeting RNA isoform specific events in cancer cells. Three key strategies for precision-based, isoform-specific targeting include: (1) Isoform Imbalance, (2) Dysregulated Splicing, and (3) Cell-type Specific Isoform Expression which can reveal unique vulnerabilities in tumor cells. These splicing related mechanisms can be leveraged for precision-based medicines, including Anti-Sense Oligonucleotides (ASOs) to correct splicing defects, cancer vaccines, antibody based immunotherapies or CAR-T cells for targeting tumor-specific neo-antigens found on the cell-surface derived from aberrant transcripts. Created in BioRender. Byrne, A. (2026) https://BioRender.com/tpk1ao9.

In addition to the altered functions, aberrantly spliced isoforms can be translated and presented on the surface of cells as neo-antigens—tumor-specific peptides that can be targeted by the immune system or cancer vaccines, even in tumors with low mutational burdens (20, 28). Identifying unique splicing signatures that occur in cancer types can be informative not only for patient stratification but also for the development of therapeutic strategies. For example, one can look at intron retention (IR) events to predict tumor-specific neoantigens (IR-neoAg). Using this approach, one group was able to find IR-neoAg events that predicted favorable outcomes in pancreatic cancer patients when given immune checkpoint inhibitors (29), indicating IR-neoAg load might serve as a good proxy for patient stratification. These unique, cancer-specific isoforms are highly attractive for therapeutic development, as they represent a rich source of neo-antigens and target specificity (**Figure 3**). In addition, scLRS allows neoantigen detection paired with the ability to look directly at heterogeneity of neoantigen expression directly in the tumor cells. Most current neoantigen-centric work uses bulk data to estimate neoantigen expression, which is unable to represent the true heterogeneity of tumor cells.

While most predictive neoantigen pipelines (e.g., ScanNeo2, Seq2Neo and nextNEOpi) are traditionally optimized for short-read data, we anticipate using long-read aware tools for variant and HLA calling, could be incorporated to support scLRS-derived targets through a structured integration framework (30–32). scLRS-derived novel splice junctions could be predicted using these neoantigen pipelines and full-length isoforms can be converted into peptide formats compatible with MHC-binding prediction algorithms using tools like NetMHCpan (33). Previous long-read studies have shown that HLA-typing can be inferred using tools like HLAminer (34). Once these peptide predictions are generated, the barcode information can be mapped back to cell-types. For fusion and splicing-derived neoantigens, scLRS provides a unique advantage by confirming whether fusions occur in-frame across the entire transcript, a critical requirement for neoantigen production that short-reads often fail to verify.

One limitation in scLRS experiments is the presence of artifacts, making identifying true isoforms difficult. These include reverse transcription artifacts, internal priming, chimeric reads and incomplete reads. Although some bioinformatic tools can remove these unwanted artifacts and recover true full-length molecules, understanding what is transcriptional noise versus protein dysfunction requires orthogonal data. For instance scLRS-detected junctions (including intron retention) can be cross-referenced against bulk short-read ‘pileups’ to confirm the precise canonical or non-canonical splice sites. Additionally, raw long-read alignments can also be validated by carefully visualizing the reads to ensure no artifacts. However, to determine the significance of these neo-antigens and to validate aberrantly spliced transcripts as productive proteins one must rely on proteomic tools.

One attractive strategy for validating novel or aberrant isoforms in cancer cells could be applying single-cell Mass Spectroscopy (scMS) experiments. Still in its infancy, scMS technology can only quantify 1000–5000 proteins across cells depending on the instrument, thus the main challenge is determining whether novel or aberrantly spliced isoforms in cancer are abundant enough to be detected using such a technique (35). Another caveat to scMS is that it suffers from lower throughput compared to sequencing based methods and is only capable of analyzing ~100 cells per experiment. Overall, most labs lack the specialty and instrumentation necessary to perform scMS and have instead applied bulk proteomic analysis in combination with long-read sequencing approaches, which may be more sensitive to novel peptides than scMS (26, 35). To date, the only study to have integrated scLRS experiments with proteomic validation using mass spectrometry data is the isoform atlas of colorectal cancer study (36). In this study they identified 394 dysregulated transcript structures in tumor epithelial cells, including numerous novel isoforms supported by TCGA and mass spectrometry data. Crucially, by incorporating scLRS data they were able to incorporate full-length transcript information and predict complete novel open reading frames from tumor-specific transcripts. This led to the identification of recurrent neoepitopes that hold potential for the development of universal neoantigen-based cancer vaccines for colorectal cancer patients. More importantly, in addition to the identifying neo-antigens for cancer, this study highlights the ability to identify tumor-exclusive isoforms which can widen the therapeutic window. scLRS allows for the discovery of surface proteins with tumor-specific splicing events or unique exon junctions. These potent targets are ideal candidates for the development of next-generation immunotherapies, including personalized cancer vaccines and engineered T-cell receptors (TCRs) that selectively target and eliminate malignant cells, offering a significant enhancement in specificity over current approaches. Overall, this elegant study integrating scLRS and mass spectrometry data lays the groundwork for developing precision based therapies.

### 2.2 Mapping the cancer genome and tumor heterogeneity and evolution

Low tumor purity samples have been a roadblock to accurate mutation detection and tumor characterization in many sequencing experiments (37, 38). Short-read single-cell sequencing theoretically allows better resolution of tumor cells, but differentiating tumor cells from normal cells within the same tissue is difficult when such classification relies solely on gene counts (38, 39). However, scLRS provides an unprecedented ability to identify somatic variants directly at the single cell level. This capability allows identification of driver variants in previously intractable samples.

Many cancer types are primarily driven by genomic structural variation (SVs) instead of SNVs (40). Therefore, to sensitively understand genotype evolution in these tumor types, a single-cell method must be able to detect these structural variants. Long-read sequencing has been previously shown to have higher accuracy and sensitivity for detecting SVs than short-read sequencing, both in DNA and RNA (41, 42). Gene fusions (when a structural variant leads to fragments of two genes expressed under a single promoter) can be strong cancer drivers and drug targets. They are also the class of SVs with the best tools for detection from long read sequencing. Two different bioinformatic tools, JAFFAL and ctat-LR-fusion, have shown the efficacy of scLRS for detecting gene fusions in cancer cells (15, 43). ctat-LR-fusion has been shown to slightly outperform other fusion callers on a standard benchmark of simulated data, but no comprehensive testing of long-read RNA fusion detection tools has yet been performed (15). Using JAFFAL, researchers showed the efficacy of scLRS for identifying different cell lines from an artificial mixture model. In one of the cell lines, they were able to identify 6 different previously validated fusions at the single-cell level. Fusion detection with ctat-LR-fusion in a T-cell infiltrated melanoma tumor sample containing only 10% tumor cells was able to identify a tumor-specific fusion event (15). Given the low fraction of tumor cells, and the low clonal fraction of the fusion, this event would most likely have been missed in bulk sequencing.

Using SNV calling, scLRS has identified cancer cell populations and their driver mutations in low-purity tumor samples (12, 13, 44). Groups have used scLRS to identify tumor-specific SNVs (13, 45) and gene fusions (15). Additionally, LongSom, a bioinformatic tool that uses variant calling on scLRS to identify tumor populations, has shown improved cell classification compared to a marker gene expression-based method (46). Tumor heterogeneity is more nuanced than just separating tumor cells from normal cells. Cancers are constantly evolving and gaining new alterations, especially when subjected to selective pressure such as drug treatment. scLRS offers an unprecedented window on tumor evolution through the ability to distinguish subclonal events within the tumor cell population. Using LongSom, the group also identified two subclones of a tumor with distinct groups of somatic SNVs and differential isoform usage (46). The ability to integrate variant calling with splicing analysis could also allow researchers to identify tumor-specific splicing that is driven directly by tumor-specific variants, increasing confidence for neoantigen selection.

scLRS has also been applied in evaluating SNVs and their direct consequences on transcript structure and/or abundance within specific cell types (44–46). scLRS studies that use genotype information include enrichment techniques such as GoT-splice, which amplifies locus-specific regions from cDNA, scTaILoR-seq, a probe-based capture approach, and scNanoGPS, a computational method designed to improve variant calling information (13, 44, 47). By improving coverage these techniques provide unprecedented ability to identify and investigate SNVs at the single-cell level. However, as with all RNA-seq approaches, they are limited in linking genotype information with splicing alterations; they infer genetic information at the cDNA level rather than capturing the full genomic sequence including introns.

### 2.3 Single-cell long-read WGS to identify tumor heterogeneity

To further understand how genomic alterations can impact transcript abundance, single-cell genome sequencing can be incorporated into assays. Single-cell whole genome sequencing (scWGS) uncovers the genetic diversity at the single cell level, including identifying SVs, SNVs and CNVs across the entire genome. Methods such as multiple displacement amplification (MDA), linear amplification via transposon insertion (LIANTI) and multiple annealing and looping based amplification (MALBEC) have been the most widely adopted techniques using short-read technologies (48–50). Given that the primary limitations when using scWGS are generating uniform coverage and sufficient sequencing depth, the first attempt using long-reads was using SMOOTH-seq (14). This scLRS method utilizes transposition with the Tn5 enzyme to fragment genomic DNA followed by strand displacement PCR and long-read sequencing using PacBio. This method pooled 16 single cells per flow cell and obtained 80,000-0.5M reads per cell, achieving variable genome coverage (10.6 - 41.3X) when applied to a chronic myelogenous leukemia (CML) cell line, K562. SMOOTH-seq on K562 cells identified the majority of cells reporting the BCR-ABL1 fusion within the cell line and had high precision in detecting insertions, deletions, and inversions compared to bulk sequencing data. SMOOTH-seq identified copy number changes at the single cell level consistent with bulk data demonstrating that two chromosomes had distinct copy number variation in two different clones of K562 indicating the potential for this method to study distinct subclones of tumors. More recently, another scLRS method was developed using a custom droplet-based multiple displacement assay (dMDA) using the PacBio platform (51) to investigate SVs, SNVs and tandem repeats within clonally expanded CD8+ T cells. This method used a full flow cell per single cell and generated 1-3M reads per cell. The dMDA method demonstrated that less sequencing was needed for recovering more true positive SNVs and SVs at the single-cell level than standard short-read whole genome sequencing methods and could achieve 40% genome coverage with overall similar uniformity to bulk WGS. They also found that integration of short- and long-read sequencing improved the detection of tandem repeats and SVs; regions difficult to resolve with short-reads alone. However, scLRS DNA experiments require more reads per cell to generate adequate coverage across the genome, limiting the number of cells per experiment. For this reason, most investigations of subclonal variants that are not focused on noncoding alterations (promoters, enhancers, or splice sites) will likely be better served by scLRS RNA sequencing in order to recover alterations in a larger sample of the cell population.

Although not a whole genome sequencing approach, integrating long-read sequencing with scATAC-seq data can provide a genomic readout identifying genetic variants as well as alterations within the chromatin landscape in cancer cells. One group developed such an approach called scNano-ATAC-seq (52) a plate-based method that utilizes Tn5 transposase to tag accessible DNA regions not decorated by histones and proteins to infer open chromatin. Oxford Nanopore sequencing reads generated using this method showed median read lengths of around 4.5 kb, capable of identifying co-accessible regulatory regions such as enhancers and promoters in an allelic specific manner. While the scLRS methods discussed here have incorporated long-read technology to assess genome health at the single-cell level, future work will need to seamlessly combine both transcriptomic and genomic information to definitively uncover the genotype-to-phenotype mapping within cancer.

### 2.4 Characterizing the immune landscape with scLRS

The clinical success of immunotherapies, such as checkpoint inhibitors and engineered immune cells, necessitates tools to predict therapeutic efficacy. One promising approach is characterizing the immune-tumor interface via immune repertoire sequencing. Immune repertoire sequencing focuses on characterizing the vast array of antigen receptors derived from B and T lymphocytes. These cells possess the unique ability to recognize an immense variety of foreign pathogens and tumor antigens through a process called somatic recombination. This mechanism involves the shuffling of three distinct gene segments—variable (V), diversity (D), and joining (J)—to generate unique receptors. Once rearranged, these segments form the Complementary Determining Regions (CDR1, CDR2, and CDR3). The CDR3 loop, which spans the V-J or V-D-J junction, harbors the highest variability and is primarily responsible for antigen recognition and specificity.

Overall, the immune repertoire is the collective diversity of antigen receptors (BCRs and TCRs) within an individual that serves as a dynamic historical record of immune surveillance and response. In oncology, profiling this repertoire is critical for understanding the mechanisms of tumor escape, monitoring responses to immunotherapies (such as checkpoint inhibitors), and detecting minimal residual disease (53, 54). Furthermore, identifying specific tumor-reactive clones is the cornerstone of developing personalized cancer vaccines and engineered immune cell therapies like CAR-T cells. Characterizing the diversity of lymphocytes and identifying their clonal expansion provides a direct window into the interactions between the host immune system and the developing tumor.

While short-read sequencing has been instrumental in cataloging CDR3 diversity, it fails at capturing the full functional potential of immune receptors. Standard short read single-cell RNA-seq experiments can perform pairing of both the Variable Heavy (VH) and Variable Light (VL) chains, but sequencing the entire mRNA transcript from end to end can only be achieved with long-read sequencing. scLRS not only enables unambiguous pairing of the chains but also captures the CDRs, framework regions and constant regions (55)—all of which can have an effect on the efficacy, avidity, and effector functions (56, 57). Critical mutations accumulated during affinity maturation that are located outside the CDR3 region are often missed by short-read single-cell immune repertoire sequencing. Studies have shown that these mutations can have functional significance (58, 59). By spanning the constant region, scLRS identifies the specific antibody isotype (e.g., IgG1 vs. IgG4) and splice variants, which dictate the immune effector function (such as Antibody-Dependent Cellular Cytotoxicity, Opsonization or Complement Activation).

To leverage these advantages, researchers have developed long-read targeted approaches. These targeted approaches use biotinylated capture probes that hybridize to genes of interest at the cDNA level and are captured using Streptavidin-coated beads followed by elution and further amplification. Using such an approach, RAGE-seq targeted T and B cell receptor sequences to infer clonal evolution and alternative splicing (9). Additionally, scTaILoR-seq implemented a similar long-read targeted approach which combined immune receptor information with transcriptome information to not only identify T cell clones but also infer differential transcript usage (44). One current issue with long-read sequencing is the lower base accuracy, most notably observed in ONT data. As a result, identifying true somatic hypermutations in B cell repertoire data can be difficult. To resolve this issue, Volden et al. developed a method using a rolling circle amplification approach to improve base accuracy called R2C2 (60). Using this approach they were able to reconstruct paired T cell and B cell receptors within peripheral blood mononuclear cells (PBMCs) and identify isotype information, pairing, and secreted vs membrane bound isoforms within B receptors (61). Despite scLRS showing great promise in identifying full-length antibody sequences, it suffers from sequencing throughput limitations and given the vast diversity of the immune repertoire, necessitates enrichment strategies. Specialized techniques like RAGE-seq and scTaILoR-seq enhance the capture efficiency of antibody sequences at the molecular level through probe-based enrichment methods, but they do not allow for full-transcriptome analysis. Alternatively, to lessen the sequencing burden and improve coverage, immune cell subsets can be selected using cell sorting, thereby limiting the number of cells needed to be sequenced. Overall, providing this complete molecular picture of immune receptors using scLRS has the potential to accelerate the discovery of potent, tumor-specific antibodies and high-affinity BCR/TCRs, directly facilitating the development of cancer immunotherapies.

### 2.5 Spatial transcriptomics adapted for long-read sequencing

One of the most rapidly emerging fields in the last decade has been the use of spatial transcriptomic technology (ST). First developed in 2016, spatial transcriptomics was rapidly adopted in the cell biology field for its ability to overcome a critical limitation of traditional scRNA-seq: the loss of native tissue context (62). In oncology, the tumor microenvironment is highly organized, and cellular function is dictated by the physical location and interaction with the surrounding cells (e.g., immune cells, stromal cells, and cancer cells). One major benefit of performing spatial transcriptomics is avoiding tissue dissociation. For instance, certain cell types are highly sensitive to dissociation protocols such as neurons, cardiomyocytes, intestinal cells, hepatocytes, myeloid cells or cells that are anchored within the tissue matrix (63–65). ST provides a high-resolution map of transcriptional heterogeneity, enabling the accurate identification of spatially defined subpopulations, the delineation of tumor boundaries, and the characterization of complex cell-cell communication networks, all of which are crucial for understanding the full breadth of the tumor microenvironment. Elucidating this complex environment at the spatial level can help infer mechanisms related to cancer progression, metastasis, and therapy resistance (66, 67).

ST technologies include sequencing-based methods that utilize spatially barcoded arrays (i.e. 10x Genomics Visium) and *in situ* imaging-based methods (like MERFISH or Xenium). Both allow researchers to quantify gene expression while retaining the spatial coordinates within an intact tissue section. Sequencing-based ST methods like 10x Genomics Visium and Slide-seq are the most compatible for long-read applications given that the 3’-end of mRNA molecules are captured and reverse transcribed using oligo(dT) probes containing spatial barcode information. Visium and Slide-seq are conceptually similar, whereby tissue sections are fixed onto slides, stained, imaged, and permeabilized releasing RNA from the cells allowing hybridization of the 3’-end to the capture probes that are either directly affixed to the slide or bound to beads (62, 68). Following post-hybridization, cDNA is generated through reverse transcription, which is then used to prepare libraries for short- or long-read sequencing. A limitation of ST is its requirement for tissue fixation and cryopreservation, procedures that lead to RNA degradation. Nevertheless, a number of studies have successfully combined long-read sequencing with ST to spatially resolve isoform information (69, 70).

Using the 10x Visium platform Joglekar et al. applied spatial long-read sequencing (spLRS) to validate regional isoform switching events in genes *Pkm* and *Ctla* observed within the mouse brain previously discovered in their single-cell long read experiment (69). In addition to this, they revealed the important biological finding of the brain-wide coordination of the *Snap25* isoform switching event whereby *Snap25-a* to *Snap25-b* was confirmed to occur in a posterior-to-anterior gradient in the spatially mapped exons, suggesting that the microenvironment can influence brain-region-specific splicing for certain genes.

More importantly, one study showed how using spLRS significantly enhanced the understanding of tumor biology in glioblastoma (71). By mapping RNA isoform diversity directly to specific spatial locations within diffuse midline glioma-H3K27M mutant (DMG) and glioblastoma (GBM) niches they were able to identify niche-specific isoform switching events, revealing an extra layer of molecular complexity critical to tumorigenesis that short-read sequencing data failed to resolve. Furthermore, they correlated the unique splice junctions and identified potential regulatory splicing factors in patient data, linking spatial molecular profiles directly to clinical outcomes (71).

The current spLRS applications represent a nascent field with immense potential for deeper biological and clinical discovery. Future applications will likely include leveraging long-read data to infer SNVs and SVs within a spatial context, enabling the resolution of malignant cell clonal populations and their evolution across different tumor niches. Another promising avenue, as detailed in section 2.4, involves spatially mapping full-length immune repertoire data. This can help characterize the immune-tumor interface by identifying specific T and B cell clones that are actively responding to the tumor. Such insights are essential for explaining post-therapy tumor dynamics and informing new drug strategies. Additionally, given that RNA degradation is a common feature in spatial technology, further optimization in improving the capture of full-length molecules is needed for characterizing isoforms within a spatial context. Overall, spLRS highlights the importance of identifying isoform switching events within spatial niches and offers a path to further stratify tumor subtypes and refine the prediction of patient outcomes in cancer therapies.

### 2.6 Single-cell epitranscriptomics

Another critical layer of complexity in RNA metabolism and gene regulation is provided by the epitranscriptome, which encompasses numerous chemical modifications to RNA molecules. These modifications can significantly impact mRNA processing, stability, translation, and ultimately, cellular function. A diverse array of regulatory factors, including RNA-modifying proteins (often termed “writers,” “erasers,” and “readers”), catalyze, remove or recognize these modifications, many of which have been directly implicated in regulating oncogenic pathways across various cancers (72–74). Among the most widely studied epitranscriptomic marks are N6-methyladenosine (m6A), 5-methylcytosine (5mC), pseudouridine (Psu), and adenosine-to-inosine (A-to-I) editing.

A-to-I editing in particular has garnered much attention given its ability to recode proteins, alter splicing, modulate miRNA binding and affect transcript stability. Functionally, A-to-I editing is significant because inosine (I) is recognized by the ribosome as guanine (G). This base change can result in non-synonymous codon alterations, impacting protein sequence, or changes in splicing regulatory elements, thereby altering the repertoire of protein isoforms. To infer A-to-I edits, studies look for adenosine to guanine mismatches in sequencing data as inosine also gets read as a guanine during reverse transcription. A recent colorectal Atlas study combined scLRS with A-to-I editing and successfully identified tumor-specific A-to-I editing events in genes *CDK13, CYPR21* and *NEAT1* which were found to be upregulated in malignant epithelial cells compared to their normal counterparts. Majority of these events were found within the 3’ UTR of transcripts, with few sites observed within the coding regions capable of introducing codon alterations. Furthermore, this research observed distinct isoform-specific editing of *CDK13*, as well as variations in editing frequencies when comparing tumor versus normal epithelial cells (36).

Among the RNA modifications, m6A is the most abundant and has been known to contribute to various cancers (75). The technical challenge of identifying m6A in an isoform specific manner at the single cell level has recently been addressed by the m6A-isoSC-seq method (76). This method adapts the short-read DART-seq approach which utilizes a fusion protein that links the cytidine deaminase APOBEC1 with the m6A-binding YTH domain for long-read sequencing. Upon transfection, this fusion protein binds to m6A sites and induces adjacent C-to-U deamination (77). The resulting C-to-U mutations are then detected in the long-read sequencing data, effectively marking the presence of m6A. Applying the m6A-isoSC-seq method to cancer cell lines they revealed that isoforms with high frequencies of m6A modification within their coding regions often corresponded to misprocessed mRNA transcripts targeted for CDS-m6A decay. Furthermore, this scLRS study revealed that m6A modifications are often isoform-specific, with different isoforms of the same gene exhibiting distinct modification levels within the same cell (76).

Crucially, the A-to-I and m6A editing scLRS studies have highlighted a phenomenon of isoform-specific RNA editing. These findings demonstrate that different splice variants originating from the same gene can exhibit distinct and varying levels of epitranscriptomic modifications within the same single cell. This isoform-specificity adds another layer of regulatory precision. While scLRS provides unparalleled resolution, orthogonal bulk long-read methods currently remain essential for robust validation. Overall, by capturing these modifications, scLRS provides a new layer of molecular data to identify cancer-specific markers and therapeutic targets that would otherwise remain undetected.

## 3 Future applications in genome-wide perturbations for functional genomics

The rise of large-scale single-cell genetic and chemical perturbation methods, such as Perturb-seq, CROP-seq, and Drug-seq, hold tremendous promise for generating the vast, high-resolution datasets needed to train deep learning foundation models and discover novel drug targets (78–81). These predictive models aim to use genetic or chemical knockouts to infer subsequent changes in gene expression, cell states, and morphological features, offering a comprehensive view of cellular dynamics. However, these foundational studies have almost universally relied on short-read sequencing, which creates a critical blind spot: they lack the ability to identify isoform-specific changes as well as genetic alterations that might be a consequence of the perturbations.

By incorporating scLRS, researchers have the ability to overcome this inherent trade-off. Aforementioned, scLRS enables the simultaneous evaluation of full-length isoform dynamics and the presence of genetic alterations, such as SNVs, SVs, and fusion transcripts, all of which could be triggered by the perturbation. For instance, by performing chemical perturbations using drug compounds, identifying genetic alterations detected at the single-cell level can help indicate possible mechanisms into drug resistance or optimal combination therapies. One interesting avenue to investigate would be applying perturbation approaches to investigate RNA binding proteins (RBPs) and their role in cancer (82). Large-scale unbiased interactome studies have discovered > 4000 RBPs representing an untapped source of therapeutic potential (83). RNA binding proteins modulate nearly every aspect of the RNA lifecycle – processing, splicing, transport, localization, stability and translation. As a result, alterations of this RNA lifecycle can have dire consequences leading to abnormal protein phenotypes that can contribute to cancer progression (84, 85). Additionally, studies have shown that abnormal expression of RBPs occur in different cancers that regulate important tumor suppressor genes and oncogenes, highlighting the need to understand RBP dysregulation and their RNA targets. For example, frequently upregulated in cancers, hnRNPA1 has been linked to poor prognosis in breast cancer and hepatocellular carcinoma (86, 87). Applying an *in-vivo* genome wide perturbation approach one study found Stau2 (Staufen 2) an RNA binding protein was a known regulator in Acute Myeloid Leukemia (88). Importantly, these perturbations can help identify RNA targets that are regulated by RBPs and by using anti-sense oligo (ASO) based therapies could recover proper splicing and restore function of tumor suppressor genes in cancer. Overall, integrating scLRS techniques with perturbation screens could provide a more definitive, functional link between a specific knockout (e.g., of an RNA binding protein (RBP) or splicing factor) and its molecular consequence (the resulting altered splicing landscape) identifying novel therapeutic targets for oncology.

## 4 Technical challenges and future improvements

The integration of long-read sequencing with single-cell platforms offers the potential to resolve complex splicing architectures, isoform-level heterogeneity and identify genetic features at the cellular resolution. However, realizing this potential requires overcoming significant hurdles inherent to long-read technology. Key limitations include data scarcity, library preparation artifacts, sequencing errors, and algorithmic variability; here we will discuss these issues and possible solutions when encountering scLRS data (**Table 1**).

**Table 1:** Challenges and possible solutions when encountering scLRS data.

Challenge	Computational/Method Solution	Mechanism of Action	Ref
Ambiguous Splice Sites	Hybrid Alignment (e.g., FLAMESv2 (101), StringTie2 (102), FLAIRv2 (103))	Uses short reads to “snap” long reads to precise canonical splice junctions.	(105–107)
Sparse Quantification	Isosceles (91)/Bambu (89)	Uses pseudo-bulk information and probabilistic modeling to rescue rare isoforms in single cells.	(94, 96)
5’ Truncation (Artifacts)	TSS Peak Calling (e.g., SQANTI3 (90), Isosceles (91))	Validates read ends against known CAGE peaks or short-read pileups to distinguish real TSS from degradation.	(95, 96)
Abundant Transcripts	CRISPR Depletion (Wet-lab)	Physically removes uninformative cDNA (mitochondrial/ribosomal) before sequencing to increase informative depth.	(99, 100)
Abundant Transcripts	Gene Targeting	Targeting using biotinylated probes to capture genes of interest	(9, 44)
Removal of 3’ 10x artifacts	Biotinylated primers	Capturing full-length 10x molecules with correct orientation	(44, 108)
Improve base accuracy	R2C2 (Mandalorion)	Concatenation of reads for collapsing and generating read consensus	(60)
Internal polyA priming	Genomic context filtering (e.g., SQANTI3 (90), TALON (105), Bambu (89), IsoQuant (106))	Flags reads ending near genomic A-rich regions and look for PAS motif “AATAAA” signals to determine if artifactual	(94, 95, 109, 110)

### 4.1 Impact of sequencing error on UMI and cell barcode fidelity

The primary advantage of long-read sequencing lies in structural transcript resolution, however the raw error profiles can pose a challenge, particularly the prevalence of insertions and deletions (indels) making it difficult to accurately assign reads to cells and molecules. While earlier iterations of the long-read platforms relied on short-read data to facilitate cell barcode identification, recent advancements, such as PacBio’s Revio achieving >99.9% base-level accuracy after consensus calling and ONT’s Q20 chemistry with simplex basecalling yielding >99% base-level accuracy, cell barcode deconvolution no longer poses as a challenge. However, given ONT’s slightly lower accuracy compared to PacBio, UMI calling can still be particularly challenging. UMIs are critical for collapsing PCR duplicates to ensure accurate quantification, thus error rates in long reads compromise this deduplication process. Sequencing errors within the UMI sequence itself can create spurious “novel” UMIs. As a result, this can cause “UMI inflation” that artificially boosts the expression counts of detected genes. Unlike short-read data, where UMI errors are typically single-base substitutions easily corrected by graph-based adjacency (e.g., UMI-tools), the complex error patterns in long reads (including homopolymer truncations) make it computationally difficult to distinguish between a sequencing error and a genuine UMI collision.

To resolve this, modern long-read pipelines (e.g., BLAZE or scNanoGPS) employ “anchor-based” detection strategies (13, 89). Rather than searching for the barcode at a fixed position, these tools identify flanking constant regions (like the polyA tail or adapter sequences) to localize the barcode, and then apply probabilistic error correction models to recover the original cell identity despite sequence corruption. Looking forward, with ONT’s transition toward Q30+ modal accuracy and PacBio’s Revio +SPQR chemistry, long read sequencing is poised to render the computationally expensive “fuzzy matching” algorithms obsolete for cell barcode and UMI deconvolution. As raw read fidelity approaches that of short-read sequencing, standard exact-matching algorithms will become viable, significantly reducing the loss of reads due to barcode errors.

### 4.2 Algorithmic variability and transcript quantification consistency

The absence of a standardized “ground truth” for long-read isoform quantification has led to substantial variability across computational tools (90, 91). Different software packages (e.g., TALON, IsoQuant, FLAMES) rely on distinct statistical assumptions for defining validity. “Strict” filters (e.g., TALON) that require an isoform to be observed multiple times often yield lower sensitivity but higher precision, whereas probabilistic models (e.g., Bambu) may recover more rare isoforms at the risk of higher false discovery rates (90). This divergence results in low overlap when different tools are applied to identical datasets, complicating cross-study comparisons. Interestingly, transcript quantification in scLRS exhibits less inter-platform variability than bulk long-read sequencing. In bulk studies, library preparation differences (e.g., PacBio Iso-Seq vs. ONT-Direct RNA vs. ONT-cDNA-seq) drive most of the variability. In contrast, most scLRS workflows generate cDNA using a shared upstream platform (e.g., 10x Genomics Chromium) before splitting the sample for PacBio or ONT sequencing. Because the input material (cDNA) is identical, observed variation is primarily driven by sequencing depth and base-calling error profiles rather than fundamental library biases, making cross-platform integration more feasible once depth is normalized (92).

To address these inconsistencies, there is a pressing need to standardize transcript quantification. The community must move away from *ad-hoc* workflows toward established benchmarks, similar to the LRGASP (Long-read RNA-Seq Genome Annotation Assessment Project) initiatives (90). These standards will allow researchers to objectively measure the false-discovery rates of novel isoforms and ensure that quantification is comparable across different datasets, platforms, and laboratories. Although currently there are a few unpublished studies that compared the scLRS technology across platforms showcasing the limitations and providing recommendations (92, 93), capitalizing on platform stability to build standardized, consensus-driven bioinformatic pipelines will be crucial for the reproducibility and clinical adoption of scLRS.

### 4.3 Reverse transcription artifacts and isoform fidelity

Template-switching reverse transcription is a prominent feature of single-cell RNA-seq methods from droplet-based single-cell platforms (e.g., 10x Genomics) to plate-based methods (e.g. Smartseq2). These artifacts complicate computational transcript identification and quantification. During cDNA generation, single-cell RNAseq experiments use reverse transcriptase, which can inadvertently switch mRNA templates during synthesis, generating chimeric cDNA molecules that fuse two distinct transcripts. Computationally, these artifacts mimic gene fusions. While algorithms for short-read data often discard such chimeric reads, long-read pipelines must actively distinguish these technical chimeras from genuine biological fusion events, a task complicated by the high sequence homology often present at switching sites. In addition to template switching artifacts, 5’ truncations can hinder novel isoform detection. Incomplete reverse transcription is a pervasive issue where the enzyme dissociates from the RNA template before reaching the 5’ end. In scLRS data, these truncated reads are frequently misclassified as novel isoforms with alternative transcription start sites (TSS). This leads to an inflation of transcript diversity, where a single biological isoform is represented by a “ladder” of truncated fragments.

Some groups have used statistical modeling to predict RT artifacts. Advanced computational tools, such as Bambu and Isosceles, employ probabilistic machine-learning models to address this ambiguity. Rather than relying solely on reference annotation, these models evaluate the likelihood that a putative novel TSS is a true biological signal versus a degradation product (94–96). This can also be achieved by integrating sequence-intrinsic features such as the presence of canonical promoter motifs or CAGE-seq data support combined with the read coverage profile (97, 98). By modeling the expected rate of RT dropout, these tools can statistically “rescue” truncated reads, assigning them to the canonical full-length isoform rather than hallucinating new transcript models. Another artifact common to scLRS is internal oligo-dT priming which can inflate transcript quantification. These events arise when the oligo-dT primer hybridizes to an exonic or intronic adenine-rich region rather than the true polyA tail of the mRNA transcript. Because these artifacts possess a polyA stretch and a UMI sequence, they cannot be removed from current scRNA-seq workflows without bioinformatic interventions. Tools like SQANTI3 take genomic context into account when identifying internal priming events (95).

Looking to the future, optimization of molecular biology procedures will be required to mitigate these artifacts. Current protocols rely heavily on template-switching reverse transcriptases that are prone to premature termination and faulty switching. Future iterations will likely need to incorporate highly processive RT enzymes or will need to combine scLRS efforts with orthogonal data like Direct RNA nanopore sequencing to truly distinguish novel transcripts from reverse transcription byproducts.

### 4.4 Throughput constraints and the “data scarcity” problem

One primary limitation of current single-cell long-read sequencing (scLRS) workflows is the finite sequencing bandwidth relative to the complexity of the transcriptome. Unlike short-read sequencing, where unique molecular identifier (UMI) saturation is often achievable, long-read platforms typically yield lower read counts per cell due to throughput and cost constraints. While long reads theoretically enable the phasing of variants and identification of SNVs on specific isoforms, the lack of depth renders *de novo* variant calling computationally difficult. Distinguishing true biological variants from stochastic sequencing errors—particularly the indels characteristic of oxford nanopore sequencing—requires much higher sequencing coverage depths (>10-20X per locus) which are rarely achieved in single-cell datasets. Consequently, rare variants are frequently indistinguishable from technical noise without aggressive data aggregation (pseudobulking), which compromises single-cell resolution. This sparsity is further magnified by the non-selective nature of current sequencing technologies. Highly abundant transcripts, such as those encoding ribosomal proteins or mitochondrial RNAs, disproportionately consume sequencing capacity, often accounting for 40-60% of the total data. This “sponge effect” reduces the effective coverage for biologically critical but low-abundance transcripts (e.g., transcription factors), imposing a variable limit of detection that confounds comparative analyses between cells with differing RNA content.

To counteract the dominance of abundant transcripts, recent protocols have integrated CRISPR-Cas9-based depletion strategies (e.g., DASH or similar methods adapted for single-cell cDNA) (99–101). By designing guide RNAs targeting non-informative, high-abundance sequences (e.g., MALAT1, mitochondrial rRNA, or ribosomal protein transcripts), researchers can selectively cleave these cDNA molecules prior to generating long-read sequencing libraries. Since sequencing adaptors are typically ligated or incorporated during PCR to the ends of intact cDNA, the cleaved fragments are excluded from the final library, effectively reallocating sequencing throughput to lower-abundance, biologically informative isoforms.

Ultimately, addressing sequencing depth and throughput is critical to call rare variants *de novo* and identify rare subclonal populations with statistical confidence. PacBio has recently elevated their throughput capabilities using the MAS-ISO-Seq/Kinnex method (102). By serially ligating multiple cDNA molecules into a single concatemer, researchers can increase the effective throughput of a sequencing run by 10- to 16-fold with up to ~80M reads per flow cell which is not far behind ONTs ~120M per flow cell. Even these sequencing depths are a far cry from Illumina’s 30B reads achieved using the X Plus system. Thus, it is important that long-read technologies continue to improve sequencing throughput, allowing sufficient depth to detect rare isoforms and genetic variants without prohibitive costs.

## 5 Conclusion and outlook

**Figure 4:**
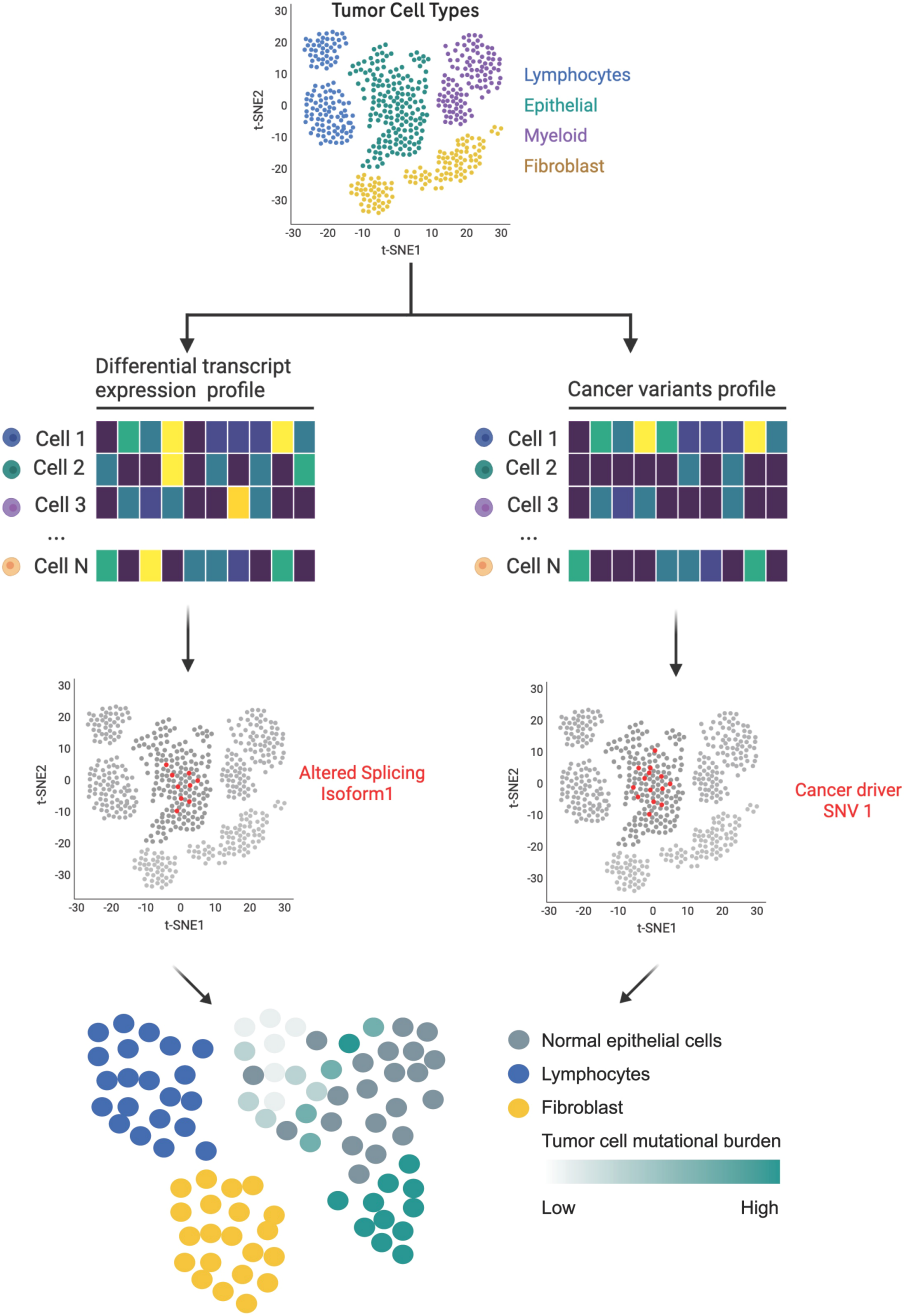
Single-cell long-read sequencing (scLRS) enhances tumor cell resolution by integrating methods for detecting splicing alterations and genetic variants. Schematic representation of how scLRS can visualize and combine multiple information types within the same cells to improve tumor cell resolution. Created in BioRender. Byrne, A. (2026) https://BioRender.com/mrmobm6.

Dysregulated splicing is not merely a feature of the cancer transcriptome; it is a hallmark of malignancy and a direct reflection of the overall genomic health of a cell. As this review has highlighted, using scLRS allows us to move beyond gene expression and better understand the profound genetic alterations and altered splicing dynamics that can arise in single cancer cells. This allows researchers to distinguish malignant populations from normal bystander cells within the tumor microenvironment, providing a clearer picture of the tumor architecture. scLRS allows the integration of transcriptomic and genomic alterations, providing a more granular understanding of tumor states (**Figure 4**). Integrating transcriptomic and genomic alterations can reveal novel tumor-specific features, facilitating the design of selective therapies that target vulnerabilities of malignant clones.

One major advantage to scLRS is the ability to identify novel tumor-specific un-annotated transcripts. This expansion of the proteome space can help unlock new biology and broaden the therapeutic potential, revealing unrecognized drug targets, diagnostic biomarkers, and improving targeting specificity. A meta-analysis focusing on FDA-approved small molecules for cancer treatment found that 76% of these drugs had issues with target selectivity (103). This highlights the importance of selective targeting to mitigate missed- and/or off-target effects during drug targeting. Aberrant splicing or cell-type specific splicing events identified by scLRS offers potent precision based targets for immunotherapies, cancer vaccines and ASO based therapies (**Figure 3**). Furthermore, monitoring splicing dynamics during treatment can reveal emergent mechanisms of resistance, such as isoform switching, or isoforms corresponding to altered or truncated proteins that evade cancer therapies which would be difficult to decipher when using short-read based single-cell sequencing. Another concrete opportunity for the field of precision medicine is to leverage scLRS data in generating foundation models that incorporate isoform-level information with complex tissue morphological and or histological features observed in tumors. A prime example is the epithelial-to-mesenchymal transition, where profound morphological shifts are directly driven by specific isoform switching events (e.g., *CD44* or *FGFR2* splicing) that alter the cellular state (24, 104). Because these isoform dynamics can be independent of overall gene expression changes, linking morphological features with splicing changes can potentially provide important insights into drug efficacy, resistance mechanisms and improve clinical prediction.

One key limitation of scLRS is its lower throughput, despite its significant advantage in detecting isoforms, SNVs, and fusions that short-read sequencing often misses. The number of sequencing reads required to identify these events varies depending on the specific analysis pipeline and the expression level of the gene of interest. Analysis of these events typically occurs at the pseudo-bulk level, which further reduces dependence on the read depth of individual cells. However, enhancing the utility of scLRS requires improved detection of events in a small number of cells. This is crucial because small, pre-existing, or newly emerging clones might harbor the necessary genomic alterations (e.g., gene amplifications, translocations, or epigenetic modifications) that enable survival under drug exposure. As this technology continues to mature, enhancing its ability to detect rarer events in tumor cells, scLRS is poised to become an essential tool in precision oncology.
